# Horizontal gene transfer: essentiality and evolvability in prokaryotes, and roles in evolutionary transitions

**DOI:** 10.12688/f1000research.8737.1

**Published:** 2016-07-25

**Authors:** Eugene V. Koonin

**Affiliations:** 1 National Center for Biotechnology Information, National Library of Medicine, National Institutes of Health, Bethesda, MD, USA

**Keywords:** Horizontal gene transfer, prokaryotes, evolutionary transitions, microbial evolution, statistical tree of life

## Abstract

The wide spread of gene exchange and loss in the prokaryotic world has prompted the concept of ‘lateral genomics’ to the point of an outright denial of the relevance of phylogenetic trees for evolution. However, the pronounced coherence congruence of the topologies of numerous gene trees, particularly those for (nearly) universal genes, translates into the notion of a statistical tree of life (STOL), which reflects a central trend of vertical evolution. The STOL can be employed as a framework for reconstruction of the evolutionary processes in the prokaryotic world. Quantitatively, however, horizontal gene transfer (HGT) dominates microbial evolution, with the rate of gene gain and loss being comparable to the rate of point mutations and much greater than the duplication rate. Theoretical models of evolution suggest that HGT is essential for the survival of microbial populations that otherwise deteriorate due to the Muller’s ratchet effect. Apparently, at least some bacteria and archaea evolved dedicated vehicles for gene transfer that evolved from selfish elements such as plasmids and viruses. Recent phylogenomic analyses suggest that episodes of massive HGT were pivotal for the emergence of major groups of organisms such as multiple archaeal phyla as well as eukaryotes. Similar analyses appear to indicate that, in addition to donating hundreds of genes to the emerging eukaryotic lineage, mitochondrial endosymbiosis severely curtailed HGT. These results shed new light on the routes of evolutionary transitions, but caution is due given the inherent uncertainty of deep phylogenies.

## Pervasive horizontal gene transfer in microbial evolution and the statistical tree of life

As soon as several complete bacterial and archaeal genomes were sequenced in the mid to late 1990s, comparative and phylogenomic analyses have revealed a surprising complexity of microbial genome evolution ^1– 6^. These observations can be broadly summarized in the form of three major trends: i) bacterial and archaeal genomes have dramatically different gene compositions, with only a small set of core genes being universally conserved; ii) unexpected patterns of gene sharing have been detected, in particular many genes shared between hyperthermophilic archaea and bacteria; and iii) topologies of the phylogenetic trees for many bacterial genes were rarely fully compatible between each other or with the 16S ribosomal RNA (rRNA) tree, although at least some of these trees were highly reliable, indicating that the discrepancies could not be caused by methodological artifacts alone. Taken together, these findings appeared impossible to explain without invoking widespread horizontal gene transfer (HGT), prompting the concept of ‘lateral genomics’, which posits that the dominant process in microbial evolution is gene exchange between organisms rather than vertical descent along a tree ^4, 7– 10^. In its extreme form, lateral genomics denies the relevance of tree-like evolution and “tree thinking” in biology altogether ^11, 12^. This concept triggered an intense debate that continued for over a decade, with the entire spectrum of positions expressed forcefully, from complete dismissal of HGT as a consequential aspect of microbial evolution to an equally adamant denial of the importance of trees ^13^. A characteristic episode that might epitomize this entire extended discussion occurred in 2006. A computational pipeline that automatically produced a comprehensive ‘Tree of Life’ (TOL) from a concatenated sequence alignment of 31 universal proteins was hailed as a major advance of phylogenomics ^14^, only to be immediately debunked as a “tree of 1%”, i.e. one that (at best) accurately reflects the evolutionary history of only a small fraction of microbial genes ^15^.

Now, exactly 20 years after the comparison of complete microbial genomes became possible, where do we stand on the status of trees and HGT in microbial evolution? Not only phylogenies of individual genes but also the microbial TOL are clearly alive and apparently rather well. A remarkable testimony to the staying power of trees is the recent amendment to the microbial TOL, which now includes a major new branch discovered through metagenomics ^16^. However, the status of the TOL has changed irrevocably. Given the overwhelming evidence that the topologies of the phylogenetic trees of individual genes are rarely identical, the phylogeny of universal genes (let it be one gene, such as 16S rRNA, or multiple genes, such as those of ribosomal proteins) hardly can be considered an accurate representation of organismal evolution. The key question, then, is: does a tree of a universal gene reflect solely its own history or does it contain information on the evolution of other genes and, if so, how many genes and how much information? A phylogenomic study designed to address this question has revealed considerable orderliness among the topologies of several thousand trees in the microbial “phylogenetic forest” ^17, 18^. Specifically, the tree topologies of the (nearly) universal genes, which encode primarily translation system components (roughly, the notorious 1% of all analyzed trees), are highly consistent not only among themselves but also with trees of numerous other genes. The consensus topology of the nearly universal trees explains nearly 40% of the variance in the tree topologies across the “phylogenetic forest” ^19^. This tree-like signal of vertical inheritance is by far the strongest trend in microbial evolution because the remaining variance in tree topologies reflects the largely random gene exchange. Thus, the “tree of 1%” seems not to be a failed evolutionary hypothesis ^20, 21^ but rather an appropriate representation of the central current of microbial genome evolution, or a “statistical tree of life” (STOL) ^22^. The STOL provides the standard against which HGT can be identified—indeed, the very notion of horizontal gene flow becomes meaningless in the absence of such vertical standard—and, more generally, the framework for reconstruction of microbial genome evolution via gene gain and loss.

A more sophisticated argument against “tree thinking” has been that biased HGT, such that there exists a gradient of HGT rates from closely related to distant microbes, could mimic a tree pattern of evolutionary divergence ^23^. Subsequent simulation analyses differed with respect to whether this explanation was plausible ^24– 26^ or not ^19^ under realistic parameters of the evolutionary process. Testing this proposition depends on the subtleties of evolution modeling and is not easy.

## Rapid dynamics of microbial evolution

Numerous comparisons of microbial genomes show that even genomes of organisms that are closely related in terms of the sequence similarity between universal genes (e.g. identical 16S rRNA sequences) often substantially differ in the gene repertoires ^27, 28^. Thus, information on the evolutionary dynamics of microbial genomes can be extracted from the patterns of gene presence-absence ^29^. The prominence of the vertical evolution trend in the “forest of life” (see above) justifies the use of phylogenetic trees of universal genes (species tree, for short) as a scaffold for evolutionary reconstruction. Briefly, all the genes in the pangenome of a species or another group of microbes (i.e. the entirety of the genes represented in the available isolates of the given group ^30^) are mapped to the leaves of the species tree. This mapping is used to reconstruct the evolutionary scenario for the pangenome, i.e. the history of gene gains, losses, and duplications. Initially, the reconstruction was performed using simple, intuitive maximum parsimony methods which identify the scenario with the minimum number of events ^31– 33^. At present, the approaches of choice are based on more sophisticated maximum likelihood algorithms that employ evolutionary birth-and-death models to derive statistical estimates for the number of different genomic events associated with each branch of the species tree ^34– 37^.

Application of the maximum likelihood approach to the reconstruction of evolution for diverse groups of closely related bacteria and archaea has revealed a striking picture of genomes in turmoil ^38^. Although the rates of gene gain, loss, and duplication greatly differ across the bacterial diversity, in the most dynamic groups, several gene gains and losses occur during the time the genome accumulates, on average, one nucleotide substitution per gene. Strikingly, the most common process of genome dynamics is actually loss of genes: for most (although not all) groups of microbes, evolutionary reconstructions indicate a twofold to threefold excess of losses over gains per nucleotide substitution. In the long term, excess of gene losses obviously would lead to genome degradation and eventually extinction, and indeed such is the fate of many groups of microbes, in particular parasites and symbionts ^39, 40^. In general, however, the gradual gene loss appears to be offset by episodic, massive gene gain that might accompany the emergence of major groups of prokaryotes ^41^ (see more below on such putative bursts of innovation). The same reconstructions indicate that in all analyzed groups of microbes, the rate of gene gain exceeds the gene duplication rate by at least an order of magnitude ^38, 42^. The primary source of gene gain is HGT, which accordingly is the principal route of evolutionary innovation in bacteria and archaea.

Taken together, the reconstructions of the dynamics of microbial genome evolution show that, in the microbial world, evolution primarily occurs not via the classic Darwinian process adopted by the Modern Synthesis of Evolutionary Biology, i.e. gradual accumulation of numerous, “infinitesimally small” changes (mutations) ^43, 44^, but rather by much bigger, at least gene-sized, leaps. In a sharp contrast to eukaryotes, in bacteria and archaea, the dominant feature of genome evolution is not gene duplication ^45– 47^ but rather evolution by extensive gene loss and gene gain via HGT.

## Essentiality and evolvability of horizontal gene transfer in bacteria and archaea

Can microbes evolve without substantial HGT, simply via the competition of clonal populations? Apparently not, as population genetic modeling indicates that this evolutionary regime is unsustainable in the long term ^48^. Clonal populations typically deteriorate due to the action of the evolutionary mechanism known as Muller’s ratchet, i.e. gradual loss of fitness and eventual extinction caused by accumulation of slightly deleterious mutations via genetic drift ^49, 50^. Demise caused by Muller’s ratchet appears to be the typical fate of bacteria that are confined to intracellular parasitism or symbiosis, although the ratchet can be slowed down by lowering the mutation rate ^51^. However, such mechanisms cannot stop the ratchet altogether. The only route of actual escape from Muller’s ratchet appears to be gene acquisition via HGT resulting in either displacement of a mutated gene by a functional copy or gain of new genes that offsets the deleterious effects of accumulating mutations ^48^. Notably, the model shows that, thanks to the stochastic nature of the mutation process, protection from the ratchet is achievable despite the fact that environmental DNA that comes from dead microbes on average has a higher mutational load than the DNA of living recipient cells ^48^. Thus, in prokaryotes, HGT plays the same role of preventing mutational meltdown that in eukaryotes is played by meiotic sex ^52^.

Escape from Muller’s ratchet can be considered the most fundamental benefit of HGT in microbial evolution but it certainly is not the only one. Acquisition of new genes and whole operons appears to be the principal route of metabolic network expansion in microbes ^42, 53^. As the network grows, gain of a single enzyme is increasingly likely to provide access to a new nutrient leading to increased fitness ^54^.

Given the indispensability of HGT for the survival of microbial populations, a plausible hypothesis seems to be that HGT is evolvable, i.e. is an adaptive, selectable trait. However, whether or not this is the case is not an easy question because HGT might be considered a by-product of the presence of substantial amounts of DNA in the environment combined with genetic processes such as transformation and bacteriophage infection that leads to gene transduction ^55^. Diverse bacteria and archaea are competent for natural transformation that is mediated by dedicated DNA intake pumps ^56^. These pumps can be legitimately considered devices for utilization of environmental DNA as a source of nucleotides (simply put, food), with HGT being a fringe benefit. However, the demonstration that at least in some bacteria the ingested DNA is protected against degradation, thus preventing its use as a nucleotide source and conversely facilitating HGT, implies that, at least in part, natural competence evolved as a gene transfer mechanism ^57^. The long-known existence of DNA uptake signal sequences and proteins that bind them, which jointly comprise a discrimination mechanism allowing bacteria to preferentially take up DNA from closely related organisms, is another piece of evidence in support of the view of transformation as an evolved route of gene transfer, apart from the nutritional value of DNA ^58, 59^.

Bacterial and archaeal conjugation (prokaryotic sex) is a mechanism of genetic material transfer between microbial cells that combines features of selfish genetic elements and devices for gene transfer. Conjugative plasmids encode proteins required for autonomous replication, whereas integrative and conjugative elements (ICEs, or conjugative transposons) typically replicate while integrated into the host chromosome but have the ability to excise and form plasmid-like molecules ^60, 61^. Both types of elements are transferred by the conjugation molecular machinery (type IV secretion systems) and typically carry ‘cargo’ genes unrelated to the transposon life cycle. Thus, these selfish elements are at the same time vehicles for HGT that mediate microbial adaptation by introducing new genes into the recipient genomes ^62^.

Perhaps the most striking showcase for dedicated vehicles of HGT are the gene transfer agents (GTAs), defective prophages that form virus particles in which they package apparently random fragments of the bacterial chromosome rather than the phage genome ^63, 64^. The GTAs then infect other bacteria or archaea, and the transferred DNA integrates into the recipient genome. In marine bacterial communities, the rate of GTA-mediated gene transfer appears to be quite high and often involves distantly related organisms ^65^. Notably, the GTAs confer on their carriers the ability to donate rather than acquire genetic material. Such a capacity could be adaptive in the context of utilization of “public goods” by microbial communities. The wide spread of GTAs appears to present strong evidence of evolvability of HGT.

**Figure 1.**
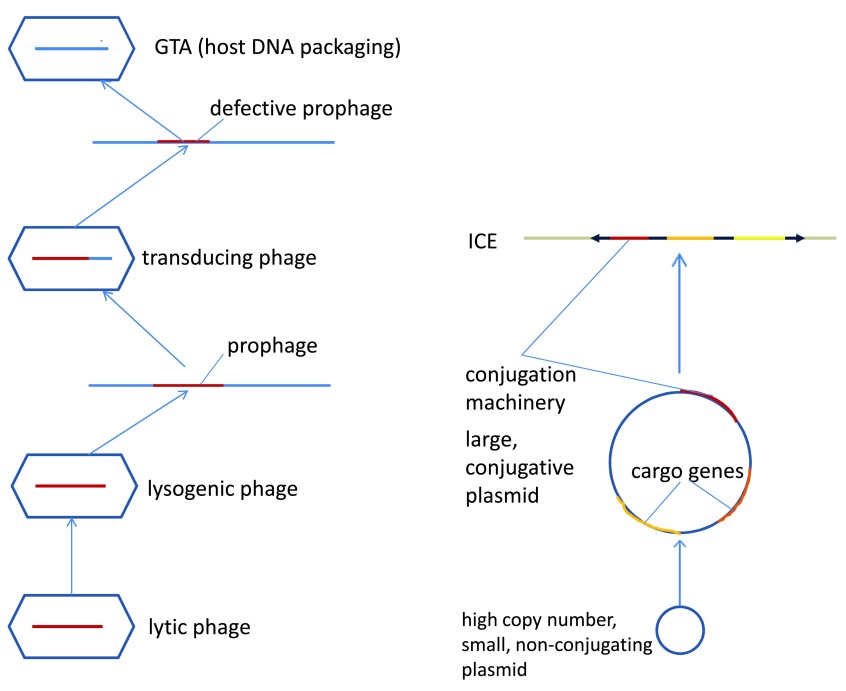
Domestication of selfish genetic elements en route to dedicated vehicles for horizontal gene transfer. The figure depicts the hypothetical stages of the evolutionary paths from a lytic phage to a gene transfer agent and from a small, high copy number plasmid to an integrative and conjugative element. Abbreviations: GTA, gene transfer agent; ICE, integrative and conjugative element.

These examples highlight the apparent major route of evolution of HGT vehicles, through stepwise domestication and “enslavement” of selfish genetic elements, such as plasmids and viruses, whereby the hosts exploit the inherent ability of such elements to transfer genetic material ( Figure 1).

## Horizontal gene transfer and evolutionary transitions

Several large-scale reconstructions of microbial genome evolution suggest that gene loss occurs in a roughly clock-like manner whereas gene gain tends to be episodic, occurring in bursts that involve acquisition of many genes over a short time ^31, 38, 66^. These observations prompted the hypothesis that emergence of new major groups of organisms often, perhaps typically, involves massive gene gain via HGT (in microbes) or extensive, in some cases, whole genome duplication (in eukaryotes) followed by gradual genome streamlining via gene loss in each of the lineages ^41^. The importance of massive HGT in at least two major evolutionary transitions, namely the origin of eukaryotes and the origin of the eukaryotic supergroup *Archaeplastida* (algae and plants), is beyond doubt ^67^. In these special cases, the sources of the hundreds if not thousands of transferred genes were mitochondria and chloroplasts, respectively, i.e. bacterial endosymbionts on the path to becoming eukaryotic organelles ^68– 70^. Can the model of punctuated gene gain be validated in a more general context? Comprehensive search of archaeal genomes for acquired bacterial genes has led to the conclusion that the origin of most, if not all, major archaeal clades was associated with and possibly caused by acquisition of hundreds or even thousands of bacterial genes ^71, 72^. The largest influx of bacterial genes was detected in mesophilic archaeal groups such as *Halobacteria* and *Methanobacteria* and apparently led to fundamental innovation, i.e. adaptation to new lifestyles and ecological niches. The conclusion on the acquisition of numerous bacterial genes at the roots of the major archaeal clades, as opposed to more uniform gain along the respective evolutionary lineages, has been reached by Nelson-Sathi and colleagues using an original statistical procedure for topological comparison of the phylogenetic tree of the (candidate) acquired bacterial genes and resident genes in the recipient group of archaea ^72^. A re-evaluation of these results using more traditional methods for reconstruction of gene gain and loss yielded results that were better compatible with piecemeal gene acquisition ^73^. Nevertheless, a more biologically oriented analysis seems to suggest that, at least for the origin of several groups of mesophilic archaea, acquisition of multiple bacterial genes has been the trigger of the lifestyle transition ^74^. Clearly, additional and probably extensive research with different methods is required to resolve this conundrum.

Two more recent, complementary studies have further addressed the question of episodic vs. continuous acquisition of genes via HGT in the context of symbiogenesis and early evolution of eukaryotes. One of these employed comprehensive comparison of the topologies of phylogenetic trees of eukaryotic genes of apparent bacterial and archaeal origin and arrived to the conclusion that eukaryotes acquired the majority of bacterial genes in the two major bursts associated with the origin of mitochondria and chloroplasts whereas subsequent, continuous acquisition of bacterial genes was limited in extent ^75^. The other analysis makes an attempt to go even further by directly comparing the phylogenetic distances from the closest bacterial homologs for the genes of apparent α-proteobacterial origin encoding proteins localized to the mitochondria and genes apparently derived from other bacteria ^76^. The conclusion is that the proto-mitochondrial genes are the closest homologs to their bacterial ancestors, hence probably the latest large bunch of bacterial genes acquired by eukaryotes.

**Figure 2.**
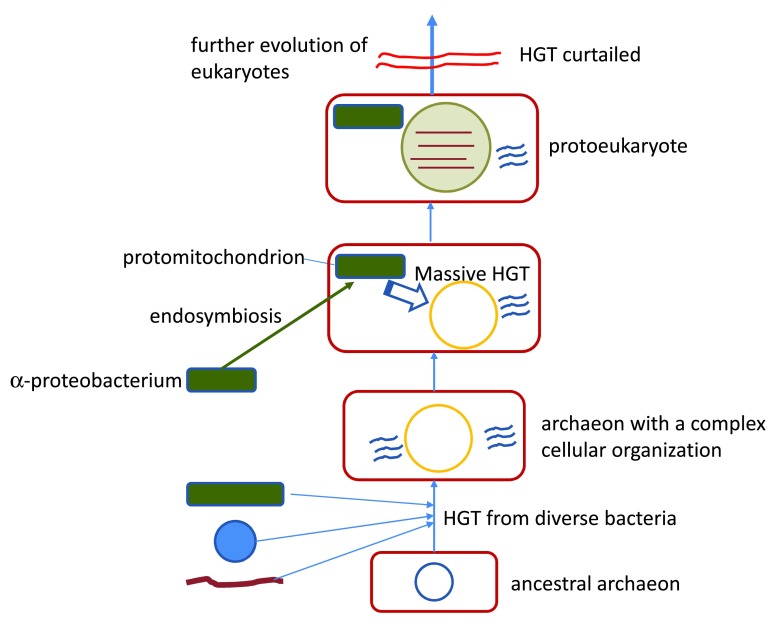
Eukaryogenesis and horizontal gene transfer. The figure presents the ‘endosymbiotic’ model of eukaryogenesis under which the host of the protomitochondrial endosymbiont was a typical archaeon albeit one with a relatively complex intracellular organization and numerous genes captured from bacteria via HGT. Abbreviations: HGT, horizontal gene transfer.

The ‘mitochondria late’ conclusion superficially could be interpreted as an indication that the host of the proto-mitochondrion was an ‘archezoan’, a primitive amitochondrial eukaryote ^77, 78^. However, this does not appear to be a necessary implication of the actual observations. On the contrary, these findings seem to be fully compatible with the conclusions of Ku *et al*. on the scarcity of late, non-organellar HGT in eukaryotes ^75^ and with the earlier scenarios of eukaryogenesis, which proposed a complex archaeon with many acquired bacterial genes as the host of the proto-mitochondria ^79, 80^ ( Figure 2). Under this scenario, acquisition of the mitochondria precipitated the series of dramatic changes in the organization of the chimeric cell which led to the curtailment of HGT. Thus, the acquisition of numerous genes from the proto-mitochondrion comes across as the last major burst of HGT (other than the acquisition of chloroplast genes at the base of the *Archaeplastida*), although numerous lineage-specific acquisitions of relatively small but biologically consequential groups of bacterial genes as well as eukaryote-to-eukaryote transfers undoubtedly occurred at later stages ^81– 83^.

## Concluding remarks

Over a decade ago, the question has been asked whether the concept of HGT would soon ‘come of age’, causing a rather tense discussion ^84, 85^. These days, I believe, it is clear that the field has matured. There is no reasonable doubt anymore that HGT is a dominant process in microbial evolution that generally occurs at a high rate. Moreover, the relevance of ‘horizontal’ as applied to gene flow is validated by the strong evidence of the existence of a central vertical, tree-like trend in genome evolution. Thus, the focus of research has shifted towards the ‘how’s’ and ‘why’s’ of HGT and, in these directions, much more remains to be done than has been accomplished already.

Both theoretical models and tantalizing experimental clues suggest that HGT is essential for microbial survival and could be an evolvable, adaptive capacity mediated by dedicated vehicles originating from domesticated selfish elements. Yet this concept runs afoul of the distrust of ‘evolution of evolvability’ that is deeply ingrained among biologists. Indeed, much work remains to be done to make a compelling case for the evolvability of HGT. Somewhat similarly, albeit in a different area, there is accumulating evidence of a major role of HGT in evolutionary transitions. Yet these conclusions rest on the analysis of deep phylogenies which are inherently error prone and recalcitrant to definitive interpretation. Much like evolution itself, extensive HGT in the microbial world is a fact and not a ‘theory’. However, understanding the routes, causes, and consequences of horizontal gene flow as well as constructing the actual, quantitative theoretical framework of this pervasive process will keep many biologists busy for decades to come.
